# Developing and Evaluating Digital Public Health Interventions Using the Digital Public Health Framework DigiPHrame: A Framework Development Study

**DOI:** 10.2196/54269

**Published:** 2024-09-12

**Authors:** Tina Jahnel, Chen-Chia Pan, Núria Pedros Barnils, Saskia Muellmann, Merle Freye, Hans-Henrik Dassow, Oliver Lange, Anke V Reinschluessel, Wolf Rogowski, Ansgar Gerhardus

**Affiliations:** 1 Department of Health Services Research Institute for Public Health and Nursing Research University of Bremen Bremen Germany; 2 Leibniz ScienceCampus Digital Public Health Bremen Germany; 3 Department of Prevention and Health Promotion Institute for Public Health and Nursing Research University of Bremen Bremen Germany; 4 Leibniz Institute for Prevention Research and Epidemiology Bremen Germany; 5 Institute for Information, Health and Medical Law University of Bremen Bremen Germany; 6 Institute for Philosophy University of Bremen Bremen Germany; 7 Department of Health Care Management Institute for Public Health and Nursing Research University of Bremen Bremen Germany; 8 Digital Media Lab University of Bremen Bremen Germany; 9 Human-Computer Interaction Group University of Konstanz Konstanz Germany; 10 Department for Health Services Research Institute for Public Health and Nursing Research University of Bremen Bremen Germany

**Keywords:** digital public health, DiPH, digital health, public health, framework, development, evaluation, guidance, program evaluation, telemedicine, intervention, technological advancement, opportunity, technology, implementation, digital public health framework, DigiPHrame, scoping review, COVID-19, contact-tracing app, contact tracing

## Abstract

**Background:**

Digital public health (DiPH) interventions may help us tackle substantial public health challenges and reach historically underserved populations, in addition to presenting valuable opportunities to improve and complement existing services. However, DiPH interventions are often triggered through technological advancements and opportunities rather than public health needs. To develop and evaluate interventions designed to serve public health needs, a comprehensive framework is needed that systematically covers all aspects with relevance for public health. This includes considering the complexity of the technology, the context in which the technology is supposed to operate, its implementation, and its effects on public health, including ethical, legal, and social aspects.

**Objective:**

We aimed to develop such a DiPH framework with a comprehensive list of core principles to be considered throughout the development and evaluation process of any DiPH intervention.

**Methods:**

The resulting digital public health framework (DigiPHrame) was based on a scoping review of existing digital health and public health frameworks. After extracting all assessment criteria from these frameworks, we clustered the criteria. During a series of multidisciplinary meetings with experts from the Leibniz ScienceCampus Digital Public Health, we restructured each domain to represent the complexity of DiPH. In this paper, we used a COVID-19 contact–tracing app as a use case to illustrate how DigiPHrame may be applied to assess DiPH interventions.

**Results:**

The current version of DigiPHrame consists of 182 questions nested under 12 domains. Domain 1 describes the current status of health needs and existing interventions; domains 2 and 3, the DiPH technology under assessment and aspects related to human-computer interaction, respectively; domains 4 and 5, structural and process aspects, respectively; and domains 6-12, contextual conditions and the outcomes of the DiPH intervention from broad perspectives. In the CWA use case, a number of questions relevant during its development but also important for assessors once the CWA was available were highlighted.

**Conclusions:**

DigiPHrame is a comprehensive framework for the development and assessment of digital technologies designed for public health purposes. It is a living framework and will, therefore, be updated regularly and as new public health needs and technological advancements emerge.

## Introduction

### Background

The overarching goal of public health is to promote and improve the health and well-being of people and communities. In recent years, digital interventions specifically designed for public health purposes have emerged on a large scale. Digital public health (DiPH) interventions may help us tackle substantial public health challenges, including aging populations [1], the dual burden of noncommunicable and communicable diseases [2], and the health impacts of climate change [3]. Moreover, DiPH interventions present valuable opportunities to improve and complement existing health care services and reach historically underserved populations.

With the COVID-19 pandemic, we have seen how digital technologies may accelerate responses to public health emergencies. For example, digital contact-tracing apps have become a major component to monitor community transmission and curb the spread of the virus in a population [4]. Further, the development of information platforms for international real-time public health data has supported policy and decision makers in planning and executing containment strategies. Another relevant field that became more visible during the pandemic concerns public health education. Digital platforms of health authorities and national agencies played a critical role in rapidly engaging and educating the population through prompt dissemination of trusted and tailored public health information, while limiting the visibility of information from unreliable sources [5].

As with other health technologies, DiPH interventions need to be developed through an iterative process, considering a multitude of factors right from the beginning of the conceptualization process. However, these factors (eg, acceptability, usability, data security, and sustainability) are sometimes not well thought out during the development or not at all considered, often resulting in low-value interventions that are ineffective and burdensome and reduce both quality and efficiency. In turn, the development of DiPH interventions is often triggered through technological advancements (ie, what is possible) rather than current public health needs [6].

Although vast amounts of new health apps are launched in app stores regularly, the number of downloads for many of these apps generally stays notoriously low [7]. Individual decisions around the initial use, adoption, rejection, and continued use of an app might be influenced by concerns regarding data security and data protection issues, costs to purchase the app, or user-friendliness for different user groups [8,9]. Other societal aspects, such as sustainable financing and regulatory requirements, are described as challenges to fulfill public health functions. Thus, these aspects may influence the design of a DiPH intervention and need to be considered from the beginning of the development process [10].

During the development and evaluation process, a number of different stakeholders assess the potential impact of DiPH interventions (eg, tech companies, health insurances, governments, and health organizations). As such, for each DiPH intervention, a great variety of potential users and user environments must be considered. To systematically develop and evaluate DiPH interventions, a comprehensive framework is needed that systematically covers all aspects with relevance for public health. This includes considering the complexity of the technology, the context in which the technology is supposed to operate, its implementation, and its effects on public health (eg, ethical, legal, and social aspects). Such a comprehensive framework would cover all phases, from conceptualization to evaluation, of all types of DiPH interventions and all parties [11].

### Existing Frameworks for Digital Health Interventions, Health Technologies, and Public Health Interventions

Interventions are often developed without a systematic method and without drawing on the evidence and theories. This point was made by Martin Eccles, Emeritus Professor of Clinical Effectiveness in the United Kingdom, in referring to a frequently used principle of intervention design, the ISLAGIATT (It Seemed Like A Good Idea At The Time!) principle. This means that we jump straight to intervention and crucially miss out understanding the behaviors we are trying to change or do not consider contextual facilitators and barriers for a successful implementation of the intervention. Frameworks that integrate a wide range of domains allow us to think ahead and help us avoid potential pitfalls before they occur so that we can design appropriate interventions based on this analysis [12].

Although frameworks for digital health interventions, health technologies, and public health interventions have been developed previously, to the best of our knowledge, no framework for the systematic development and assessment of digital interventions for public health purposes exists today. Assessment criteria for health-related technologies have been developed previously, although their focus generally lies on either health technology [13,14] or digital health-relevant [15] aspects.

One prominent example of assessing various health technologies is health technology assessment (HTA). “HTA is a multidisciplinary process that uses explicit methods to determine the value of a health technology at different points in its lifecycle. The purpose is to inform decision-making in order to promote an equitable, efficient, and high-quality health system” [16]. Based on this methodology, various organizations have developed frameworks with different foci [13,14,17]. For instance, the European Network for Health Technology Assessment (EUnetHTA) developed the health technology core model for assessing the dimensions of value to facilitate the production and sharing of HTA information, such as evidence on efficacy, effectiveness, and patient aspects, to inform decisions. The model has a broad scope and offers a common ground to various stakeholders by offering a standard structure and a transparent set of proposed HTA questions [13]. HTA frameworks are generally applied to already developed technologies rather than providing standards for evaluation aspects that should be considered throughout development. However, this is important because existing interventions would likely be outdated by the time their assessment is finished.

Assessment frameworks specifically designed for the evaluation of digital health technology also exist. The National Institute for Health and Care Excellence (NICE) recently developed an Evidence Standards Framework (ESF) for digital health technologies [15], aiming at providing standards for clinical evidence of (novel) health technology’s (cost-)effectiveness within the UK health and care system. Similar to other frameworks [18-21], the ESF lacks applicability to public health technologies, due to its focus on clinical outcomes. Other frameworks focus on evaluation and assessment criteria along the life cycle of digital health interventions yet still lacking a public health focus [22].

Digital interventions heavily rely on user interaction and engagement. However, public health frameworks generally do not include specific measures to assess usability, user experience, and the design aspects crucial for promoting sustained user engagement [23]. Furthermore, DiPH interventions often require integration into existing health care systems, which can be complex and fraught with interoperability, data security, and data protection challenges—issues that are often not properly addressed in public health frameworks [24]. Although these are just a few examples, they illustrate how unique aspects of DiPH interventions may fall short in existing public health frameworks.

### Gaps and Objective

Together, we identified the following gaps:

- Absence of a framework for digital interventions in public health: Although frameworks for health technologies and public health interventions exist, there is no established framework specifically tailored for the systematic development and assessment of digital interventions in public health.
- Limited applicability of existing assessment frameworks.
- Inadequate consideration of usability and integration challenges.

Addressing these gaps requires the development of a comprehensive framework specifically tailored for digital interventions in public health, integrating diverse domains and considering usability, user experience, and integration challenges throughout the development and assessment process so that developers and assessors need not draw on multiple frameworks. The main focus of this paper was to present the current form of the digital public health framework (DigiPHrame) and describe its development process, followed by a use case to illustrate its application. More detailed information on the scoping review that served as a starting point to develop DigiPHrame can be found in the protocol that we preregistered on the Open Science Framework (OSF) [25]. The German contact-tracing app Corona-Warn-App (CWA), as a digital public warning system with a clear public health focus, was deemed as a suitable use case to illustrate the application of DigiPHrame.

## Methods

### Search Strategy

**Figure 1:**
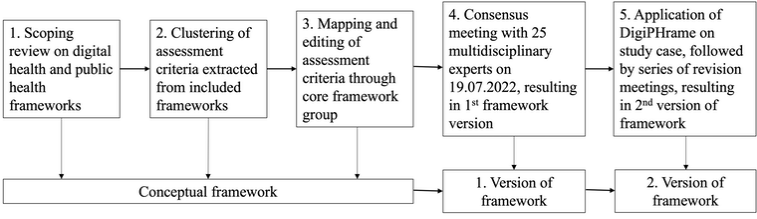
Methodological summary of the DigiPHrame development approach. DigiPHrame: digital public health framework.

We developed DigiPHrame in several steps, as shown in Figure 1. First, we conducted a scoping review to identify existing frameworks for public health and digital health interventions (see the protocol and registration on the OSF [26]). See Table 1 for the eligibility criteria. For information sources, we searched journal papers in the electronic literature databases MEDLINE (via PubMed), Scopus, IEEE, CINAHL (via EBSCO), and PsycINFO (via Ovid). Our search strategy was first developed around our core concepts as our primary search keywords and Boolean operators: (“Public Health” [Title/Abstract] OR “Digital Health” [Title/Abstract]) AND Evaluation [Title] AND Framework [Title]). The search syntax was then expanded to include the synonyms, wildcards, and relevant subject terms of the primary keywords to increase the sensitivity of our searches. Next, we modified the subject terms and search field of the search syntax to adapt to each database (see Multimedia Appendix 1). We also manually searched relevant reviews’ reference lists. The final search was completed on April 12, 2022, with no publication date limitations.

**Table 1:** Eligibility criteria for the scoping review to identify existing frameworks for public health and digital health interventions.

Criterion	Inclusion	Exclusion
Framework	Development or evaluation framework for health interventions related to public health or digital health	No framework or guidance in the report
Report	Framework or guidance outlining the standards, principles, criteria, or properties needed to support the systematic development or evaluation of health interventions aimed at health promotion or prevention with or without digital technologies	Framework or guidance not focusing on developing, monitoring, validating, or evaluating health interventions; not providing specific standards, principles, criteria, or properties; only designed for 1 specific tool; and not applicable to other health interventions and only applicable to pharmaceutical/surgical/clinical/rehabilitation interventions
Publication type	Journal papers, study/policy/program reported in gray literature	Comment, correction, letter, editorial, protocol, oral presentation, poster
Language	English	Other language than English
Access to full text	Access to full text of studies selected for data coding	No access to full text

### Selection of Sources of Evidence

After deduplication, 4830 titles and abstracts were screened by 2 researchers independently, resulting in 433 (9%) full texts, which were then assessed by 2 independent researchers. Disagreements between researchers were resolved through dialogue, with the involvement of a third party, if necessary, although a definitive agreement score was not established. In total, 68 (15.7%, see Multimedia Appendix 4) papers were included for data extraction (see the Preferred Reporting Items for Systematic reviews and Meta-Analyses [PRISMA] flowchart in Multimedia Appendix 2 [27]).

### Data Charting and Data Items

We extracted all pertinent assessment criteria from the frameworks identified through the scoping review. Initially, these criteria were assigned to HTA domains/subdomains [13], although several criteria could not be assigned due to thematic misfit (akin to deductive coding). Subsequently, new categories were formed (akin to inductive coding). One researcher performed the coding initially, followed by a collaborative examination of the coded sections by 2 researchers, leading to adjustments during the discussion process (eg, reassignment to other domains, reassignment to other subdomains within a domain, summarization of subdomains, and deletion of irrelevant domains or subdomains). Questions describing the subdomains were devised by us based on the criteria (here, too, a proposal was made by one person, followed by verification by a second person). We consulted additional literature for the categorization of ethical principles [28].

A group of multidisciplinary experts from the Leibniz ScienceCampus Digital Public Health (LSC DiPH) were assigned to the domains corresponding to their expertise for counseling. Each domain was restructured with proficient inputs to represent the complexity of DiPH. Where necessary, additional literature was consulted, especially when the included frameworks fell short of offering criteria specific to DiPH.

### The Proposed Framework

A first draft of the proposed framework was sent to an interdisciplinary expert panel consisting of 105 members of the LSC DiPH. Feedback was gathered as unrestricted comments on the domains we developed. We reached out to experts from diverse fields, including medicine, public health, global health, psychology, sociology, human-computer interaction, (health) economics, informatics, sports science, medical biometry, architecture, urban planning, statistics, ethics, policy analytics, and law, assigning them domains based on their respective expertise. A deadline for feedback submission was set for July 18, 2022. Additionally, the same members of the LSC DiPH were invited to partake in a consensus meeting held on July 19, 2022. Participants were grouped into domain-specific discussions according to their areas of expertise, with these discussions being moderated by the DigiPHrame team. This resulted in the first version of the framework [29].

### Use Case: Corona-Warn-App

Shortly after the COVID-19 pandemic in 2020, numerous digital contact-tracing apps were developed or proposed, with official government support in some territories and jurisdictions. The rationale was that contact tracing is an important tool in infectious disease control, but as the number of cases rises, time constraints make it more challenging to trace transmissions effectively. Digital contact tracing, especially if widely deployed, may be more effective than traditional methods of contact tracing [30].

COVID-19 apps include mobile apps for digital contact tracing (ie, identifying persons, or “contacts,” who may have been in contact with an infected individual) deployed during the COVID-19 pandemic. Privacy concerns have been raised, especially about systems tracking users’ geographical location. Alternatives include co-opting Bluetooth signals to log a user’s proximity to other smartphones. For example, the open source CWA funded by the German government was based on proximity tracing using Bluetooth signals. The app provides a function for users to warn other users by uploading their positive test results anonymously on a voluntary basis to the CWA server. Users are then notified about any contacts with infected persons and can get tested on a voluntary basis.

The same experts were invited to a workshop on February 23, 2023, where the proposed framework was applied to a study case and tested for face validity. The case study was the CWA. Followed by several revision meetings by the framework team between February and May 2023, the second version of the proposed framework was finalized in May 2023 [31].

### Ethical Considerations

Ethical approval is not applicable for this study since it did not involve human subjects.

## Results

### Characteristics of DigiPHrame

**Figure 2:**
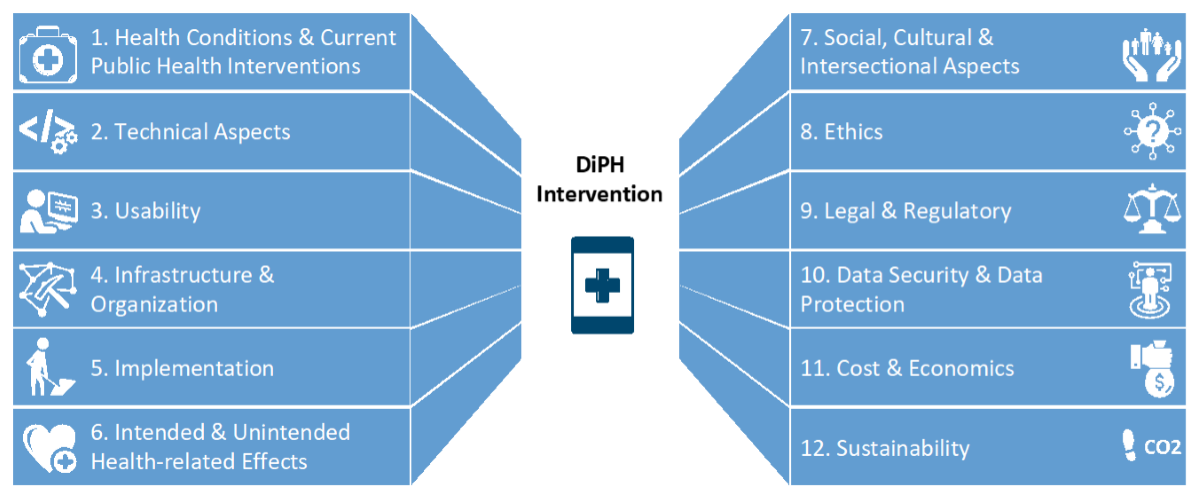
Summary of DigiPHrame domains for developing and evaluating DiPH interventions. DiPH; digital public health; DigiPHrame: digital public health framework.

DigiPHrame comprises a set of criteria framed as open-ended questions clustered within domains that lead interested parties through a broad spectrum of crucial elements when developing and evaluating DiPH interventions. The evolution of domains and subdomains through the stepwise process, including the number of questions per subdomain in each version, can be found in Multimedia Appendix 3. The framework in its current form was uploaded to the LSC DiPH website and the OSF [32] in May 2023 and is a revised version of the original framework that was first published in July 2022. In total, DigiPHrame consists of 182 questions, structured by 12 domains (Figure 2).

Domain 1 describes the current status of health needs and existing interventions; domains 2 and 3 are aimed at the DiPH technology under assessment and aspects related to human-computer interaction, respectively; domains 4 and 5 are aimed at structural and process aspects, respectively; and domains 6-12 assessment criteria address contextual conditions and the outcomes of the DiPH intervention from broad perspectives.

Next, we defined the domains and illustrated how DigiPHrame can be applied using the CWA as a use case. The CWA is a digital public warning system that was designed and developed during the COVID-19 pandemic and has a clear public health focus. We briefly outlined the purpose and characteristics of the CWA. From each domain, we applied 1 assessment question as an example.

### Use Case: Corona-Warn-App

#### Domain 1: Health Conditions and Current Public Health Interventions

Domain 1 involves background information for DiPH interventions, describing the population, conditions, and observance of health inequities. Furthermore, this domain addresses current public health interventions and common alternatives.

Question 1.5 asks, “What is the expected level of digital literacy of the target population?” In the case of the CWA, the target population comprises the entire population within a geographically delimited space. Therefore, the entire spectrum of digital literacy is to be expected. Thus, different forms of representation (eg, graphics, text, and sound) of risk exposure and other information related to the COVID-19 pandemic must be available, which was not the case and might have prevented people from using it.

#### Domain 2: Technology and Usability

Domain 2 guides one through assessing general technical aspects of the health technology of interest. The questions focus on what digital tools are applied and how aspects such as interoperability, data integration, internet connectivity, and others are integrated.

Question 2.17 is, “Does the software require an internet connection (eg, all the time, once in a while, or once)?” In the case of the CWA, an internet connection is necessary as the major functionality of warning people is distributed via the internet. Only a fraction of the available functions work completely without an internet connection, such as the contact diary. Generally, the app does not need a continuous internet connection. However, the device on which the app is installed needs to be connected to the internet, at best multiple times a day but at least once a day to sync the contacts and update on test results.

#### Domain 3: Usability

Domain 3 focuses on how usable the health technology system is in order to ensure that its users can perform the required tasks (ie, the intended function) safely, effectively, efficiently, and with satisfaction. Therefore, accessibility, user empowerment, credibility, and trustworthiness are also considered in this domain.

Question 3.3 asks, “Are the health technology and DiPH intervention available in relevant languages?” When the CWA was first launched, it was available only in German and English. Russian, 1 of the most spoken immigrant languages in Germany, was not provided in the CWA until much later versions. Since version 2.20.0 for iOS and version 2.20.4 for Android, the CWA is available in German, English, Turkish, Bulgarian, Polish, Romanian, and Ukrainian.

#### Domain 4: Infrastructure and Organization

Domain 4 on structural aspects considers the structure of the context in which a DiPH intervention is developed and implemented, as well as the involved stakeholders. Question 4.4 asks, “Is the DiPH intervention flexible to suit local, cultural, or social needs?” Initially, the German government promoted centralized storage of user data, which, according to the Federal Ministry of Health, would allow it to better track the spread of infections. However, this led to resistance from digital experts and data protectionists. As a consequence, the CWA was developed, with decentralized data collection across various servers. This approach ensured that the data could be decoupled, thereby hindering any potential tracing of app users.

#### Domain 5: Implementation

Domain 5 describes aspects to consider before and during integration of a DiPH intervention into the health care system to ensure that the intervention is delivered properly. The domain focuses on the theory used for implementing the DiPH intervention, infrastructure, process, and agents, as well as implementation outcomes and dissemination.

Question 5.9 asks, “Which implementation difficulties (eg, duration, scope, disruptivity, centrality, complexity, and the number of steps required) did the DiPH intervention encounter?” In case of the CWA, necessary features (eg, sharing test results and embedding vaccination certificates) were not initially available when the app was first launched in June 2020, but had to be continuously added to the app.

#### Contextual Conditions and Outcome-Related Domains

##### Domain 6: Intended and Unintended Health-Related Effects

Domain 6 considers the positive and negative effects of a DiPH intervention on physical, mental, and social health; the quality of life and well-being; and the knowledge, beliefs, and behavior of individuals and the population in the short, intermediate, and long terms.

Question 6.2 asks, “To what extent is the DiPH intervention expected to impact the physical, mental, and social health of the individual and the population?” With its goal to prevent infections, the CWA was expected to positively affect individuals’ and, ultimately, population health. It is unclear how the large red warning sign displayed on users’ smartphones when a high-risk contact with an infected person occurs would affect their mental health. Although generally accepted, the CWA was not used by the majority of the population and was widely discussed in terms of data privacy concerns prior to the launch of the app. With some individuals using the CWA and some not (including, sometimes, strong opinions in favor or against the benefit of the app within a social circle), this may have affected an individual’s relationships and social health.

##### Domain 7: Social, Cultural and Intersectional Aspects

Domain 7 examines the societal, cultural, and intersectional dimensions pertinent to communities and groups of individuals, such as ethnic or demographic groups, people residing in the same neighborhood, those sharing common interests, or individuals with specific physical or mental conditions.

Question 7.5 asks, “Which factors in the society/community are relevant for DiPH intervention implementation?” In the case of the CWA, it is the availability of compatible smartphones (eg, older smartphones are not compatible), trust that data will be protected and not used for other purposes (eg, analog data from guests of restaurants, not the data from the app, were used to identify suspects of thefts), and willingness to enter one’s data in the case of infection.

##### Domain 8: Ethics

Domain 8 addresses the moral considerations that arise from the implementation of DiPH interventions. The categorization of ethical principles is based on the influential *Principles of Biomedical Ethics* by Beauchamp and Childress [28].

Question 8.20 asks, “Does the DiPH intervention discriminate against particular segments of the target population?” Although efforts were successively visible to avoid discrimination, it took too much time to offer the app in different languages frequently spoken in Germany. People using phones with older operating systems were also excluded from applying the app.

##### Domain 9: Legal and Regulatory Aspects

Domain 9 generates awareness about which areas of law must be considered when developing or evaluating DiPH interventions. It is not the purpose of the domain to pose every specific legal question that has to be answered in order to develop or evaluate DiPH interventions. Since laws differ from country to country, the domain helps detect fields of law and typical problems in those fields that could be relevant for developers and evaluators. The applicable law and its requirements depend on the country.

Question 9.6 asks, “Have you considered the potential reimbursement of DiPH interventions in a national health system (some countries may have specific requirements for reimbursement)?” This raises awareness about the requirements for reimbursement of the DiPH interventions in a national health system or for other payers. Regarding the CWA, the provider offered the intervention for free (without a reimbursement option) because the free-of-charge offer of the CWA promises a broader and quicker distribution of the app.

##### Domain 10: Data Security and Data Protection

Domain 10 focuses on the technological protection of data and, therefore, combines the aspects of data confidentiality, data integrity, data authenticity, data availability, and data controllability. Data protection relates to whether the system is allowed to process personal data.

If personal data are transferred to third parties, question 10.25 asks, “Is there is a legal basis for the transfer, and are the requirements of the legal basis fulfilled?” Regarding the case of the CWA, T-Systems International GmbH and SAP Deutschland SE & Co. KG are acting on the Robert Koch Institute’s behalf. The legal basis is a contract that is binding on the processor with regard to the controller and that sets out the subject matter and duration of the processing, the nature and purpose of the processing, the type of personal data and categories of data subjects, and the obligations and rights of the controller (Art. 28(3) of the General Data Protection Regulation [GDPR]). Otherwise, the Robert Koch Institute only passes on data to third parties if it is legally obliged to do so or if this is necessary for legal action or criminal prosecution in the case of attacks on the app’s technical infrastructure.

##### Domain 11: Cost and Economics

Domain 11 assesses DiPH interventions regarding whether they can be considered a rational use of scarce resources. Question 11.1 asks, “Which relevant costs and effects can be identified?” Considering the costs and effects of a DiPH intervention from the beginning could help compare it with other interventions and show that it is economically dominant (ie, it is at least as effective as but costs less than the alternative interventions). Further, this information might be the basis for health economic evaluation (see questions 11.4- 11.6) to see whether what it costs per health gain is considered acceptable by the payer. In the case of the CWA, there are various relevant costs of the intervention itself, such as development and operation (2020: €52.8, or US $57.5, million; 2021: €63.5, or US $69.1, million) and promotion (2020 and 2021: €13.7, or US $14.9, million) [16]. Taking a broader (societal) perspective, there might be further costs, such as costs of further testing when the CWA receives a warning and costs of unrelated survival gains or benefits, such as a reduction in the loss of earnings, reduction in hospitalizations and rehabilitation measures, and reduction in deaths [32]. However, to the best of our knowledge, the pandemic context and the decision process about the CWA lead to a situation where a decision was made without formally considering cost-effectiveness in comparison with alternative decision options.

##### Domain 12: Sustainability

Domain 12 asesses environmental, social, and economic sustainabilty. Given the goal to reduce carbon emissions in health care, question 12.1 asks, “Which resources are necessary to develop and maintain the DiPH intervention?” In the case of the CWA, servers need to run, which produces carbon emissions, and computers need to be obtained to ensure compatibility of health offices with the CWA. Measuring and evaluating these resource consumptions also allows decision makers to consider more climate-friendly design alternatives for DiPH interventions.

### Application of DigiPHrame for the Development and Evaluation of DiPH Interventions

In the use case of the CWA, we highlighted a number of questions relevant during the development of the CWA but also important for assessors once the CWA was available. For example, developers needed to consider how the data would be collected and shared without interfering with data privacy and data protection laws. Similarly, assessors needed to find ways of evaluating the effectiveness of the CWA (eg, did the CWA prevent infections?) without relevant data (due to decentralized data storage, data from different individuals could not be connected, and thus, only estimates could be determined). In future scenarios, DigiPHrame can serve as a checklist for both developers and assessors to help them avoid overlooking key issues with relevance to the performance of the intervention. Although for some questions, it might be enough to use common sense (in the case of the CWA, it could be questions surrounding the usability of the app), for others, specialist expertise may be necessary (eg, questions regarding legal and regulatory issues).

DigiPHrame is agile and primarily user led (Textbox 1). We deliberately included the option of feedback loops in the framework to support the agile development process. Although it is advised to consider all domains and respective questions, developers may decide which domains are assessed at what stage of their development process and which questions are relevant for the respective DiPH intervention. For an intervention under development, a first orientation might be enough to understand whether it is worth continuing along the determined path or whether adjustments might be necessary. Developers may also decide to put specific questions on hold and revise them at a later stage in case any changes or additions need to be made to the DiPH intervention. Similarly, assessors may delay answering certain questions in case no robust evidence is available at the time to answer the questions.

**How to use the digital public health framework (DigiPHrame).**

Users of DigiPHrame are encouraged to first answer a list of questions regarding a general description of their digital public health (DiPH) intervention. Providing general characteristics will help assessors better understand the DiPH intervention under assessment. DigiPHrame is further equipped with a standardized answer scheme to help developers in answering the questions and, if necessary, plan the next steps in the development process. For assessors, the answer scheme can serve as a checklist to tick off all relevant criteria.

DigiPHrame users can respond to each question using the provided answer scheme. The first 2 assessment indicators are “not applicable” when the question is irrelevant to the particular DiPH intervention and “assessment result” to provide the answer or additional information to the assessor. The last 3 columns of the answer scheme focus on the current status of the DiPH intervention during the assessment. These columns include “Assessment completed and sufficient” when the assessment is finished and satisfactory, “Assessment done but improvement needed” when the assessment is complete but indicates the need for improvements or changes to the DiPH intervention, and “Assessment only partially done or not possible yet” when the assessment is incomplete or not feasible at the moment.

**Example answer scheme:**

Criterion: population

Question: Who is the target population of the DiPH intervention?

Assessment indicator scheme:

- Not applicable (N/A): yes
- Assessment result: The entire population is at risk of getting infected with SARS-CoV-2.
- Assessment completed and sufficient: yes
- Assessment done but improvement needed: Briefly outline the necessary changes/expected date for revising the question.
- Assessment only partially done or not possible yet: Insert specific steps to be taken/expected date of completion.

## Discussion

### Principal Findings

#### A Unified Framework for Digital Interventions With a Public Health Focus

Although health-related digital technologies hold great potential for enhancing public health and addressing health-related inequalities at a relatively low cost, new developments are often driven by technological advancements and assessments and primarily revolve around clinical aspects of health. To the best of our knowledge, no existing frameworks consider digital interventions specifically designed for public health purposes. Additionally, previous frameworks primarily emphasize clinical aspects when addressing digital health technologies, neglecting the public health perspective. As an example, although the ESF [15] emphasizes clinical outcomes, crucial for any intervention’s success, it omits essential aspects, such as sociocultural, ethical, legal, and sustainability factors, vital for effectively implementing DiPH interventions. DigiPHrame includes aspects regarding clinical outcomes (eg, domain 6: intended and unintended health-related effects), among others derived from the ESF, but also the above-mentioned factors. Moreover, although HTA frameworks [13] are often designed for evaluating existing technologies, our objective was to devise a comprehensive framework applicable across all stages of development and evaluation. DigiPHrame adopts a comprehensive public health perspective and can serve as a guide, specifically for developers and assessors throughout the entire development and assessment of DiPH interventions. DigiPHrame provides users with criteria concerning clinical effectiveness, technical functions, and usability, as well as organizational, legal, ethical, economic, and sociocultural aspects. Users have the flexibility to determine the relevant domains and assessment questions based on their specific needs and the stage of the process without relying on multiple development and assessment frameworks. Furthermore, the users of DigiPHrame are encouraged to take a broader view and may be inspired to include other perspectives that were not initially within their scope (eg, sociocultural aspects, ethics, and sustainability).

#### A Holistic Framework Covering Relevant Domains for DiPH Interventions

Additionally, a deeper understanding of contextual factors is necessary to assess what will work in one country versus another. These factors can either enhance or hinder the adoption and diffusion of DiPH technologies. Although many frameworks tend to overemphasize technical aspects, it is essential to acknowledge that various other factors influence success or failure. These factors include disparities in health expenditure, demographic conditions, health infrastructure, information and communication technology (ICT) skill levels, digital health literacy, clinical and patient engagement, and many more. Recognizing and understanding these key differences within and across countries is crucial for policy makers and other stakeholders in public health and DiPH. Although our framework considers these factors, future work needs to apply DigiPHrame in diverse contexts and countries to validate and continuously update the current version of the framework. In addition, although our framework aims to be universally applicable to various DiPH technologies, it will require revision as new public health needs and DiPH technologies emerge. Therefore, our framework can serve as the foundation for a development and assessment toolkit that developers, decision makers, and other users alike can use.

As we illustrated with the German contact-tracing app CWA, that was launched during the first wave of the COVID-19 pandemic, DigiPHrame can be applied for all stages, including design, implementation, and evaluation. This may have helped avoid potential pitfalls from the beginning that would have otherwise occurred further down the development process.

### Strengths and Limitations

Our framework has several key strengths that set it apart. First, it is based on a comprehensive scoping review of digital health and public health frameworks (OSF [25]), ensuring a robust foundation. Additionally, we conducted scientific consensus meetings involving interdisciplinary experts, ensuring a breadth of perspectives in its development. Second, the assessment themes within our framework were derived from existing frameworks developed in various Western countries, including Germany, the United Kingdom, and the United States. This demonstrates the broad applicability of DigiPHrame across different geographical contexts, making it adaptable to diverse settings in high-income countries. Another strength of our framework is its universality. It is not limited to specific types of DiPH interventions and, therefore, can be applied to any digital intervention with the overarching aim of improving public health outcomes. This flexibility allows for its widespread application across a wide range of interventions. Furthermore, DigiPHrame is designed as a living framework that will evolve and adapt as technology advances. To do so, we will continue to revise the domains and questions and regularly test any changes for face validity using a variety of use cases. This will ensure that it remains relevant and up to date in the fast-paced DiPH landscape, accommodating emerging technologies and methodologies. Lastly, we incorporated input and expertise from various research fields throughout the entire development process of DigiPHrame. We fostered an interdisciplinary perspective by involving experts from different disciplines, including public health, epidemiology, psychology, philosophy, law, economics, human-computer interaction, and sociology, enriching the framework with diverse insights and knowledge.

Although our framework has several strengths, it is important to acknowledge certain limitations. First, going through the proposed framework might require significant time and expertise due to its complexity and depth. Nevertheless, it is flexible; it is up to the assessor to decide which domains and criteria are applicable to their specific case. This flexibility is advantageous, allowing the framework to be adapted to diverse contexts and DiPH interventions. However, it may also introduce subjectivity in the evaluation process, as different assessors may choose different domains and criteria, leading to varying outcomes. Ensuring transparency and consistency in domain selection could help mitigate this concern. Additionally, we intend to develop a condensed version of the framework focusing on the most critical domains and questions. Second, we engaged experts from diverse research fields to address potential inconsistencies during the development process. However, it is worth noting that the majority of our consultations did not extend to a broader geographical range, particularly in terms of incorporating specific aspects from low- and middle-income countries. It is crucial to recognize that contexts may differ significantly, including factors such as technology accessibility, digital health literacy, and legal requirements. Although DigiPHrame aims to be applicable across different geographical contexts, users of the framework are advised to consider and adhere to their local requirements and nuances. Furthermore, in our scoping review, we focused on primary prevention and health promotion but not on secondary and tertiary prevention (eg, rehabilitation). This could have limited the frameworks and criteria we found. Although as per our definition, DiPH focusses on primary prevention and health promotion, future research may also include frameworks focused on secondary and tertiary prevention. Lastly, we did not provide any evaluation methods along with the framework. As DigiPHrame evolves, however, our goal is to provide suitable existing methods and develop novel evaluation methods for DiPH interventions.

### Conclusion

DigiPHrame is a comprehensive framework for the development and assessment of digital technologies designed for public health purposes. Our framework may assist in designing and evaluating DiPH interventions that serve public health needs rather than displaying technological advancements. Moreover, DigiPHrame may help avoid overlooking important aspects, such as acceptability, usability, data security, and sustainability, which would otherwise result in low-value interventions that are not user friendly, violate (data protection) laws, or are not sustainable. We aim to revise and improve DigiPHrame as new technologies emerge, and encourage developers and assessors to use and contribute to improving DigiPHrame.

## Appendix

Multimedia Appendix 1 Search syntax.

Multimedia Appendix 2 Preferred Reporting Items for Systematic reviews and Meta-Analyses (PRISMA) flow diagram.

Multimedia Appendix 3 Evolution of domains and subdomains.

Multimedia Appendix 4 Included Reports.

## Abbreviations

CWA: Corona-Warn-App
DiPH: digital public health
DigiPHrame: digital public health framework
ESF: Evidence Standards Framework
HTA: health technology assessment
LSC DiPH: Leibniz ScienceCampus Digital Public Health
OSF: Open Science Framework

## Data Availability

The data collected and analyzed during this study are available from the corresponding author upon reasonable request.
