# Supplementary material for: Developing and Evaluating Digital Public Health Interventions Using the Digital Public Health Framework DigiPHrame: A Framework Development Study
Source: J Med Internet Res. 2024 Sep 12;26:e54269. doi: 10.2196/54269 (PMC11427851; doi:10.2196/54269)
Supplement: Multimedia Appendix 2 [file jmir_v26i1e54269_app2.pdf]

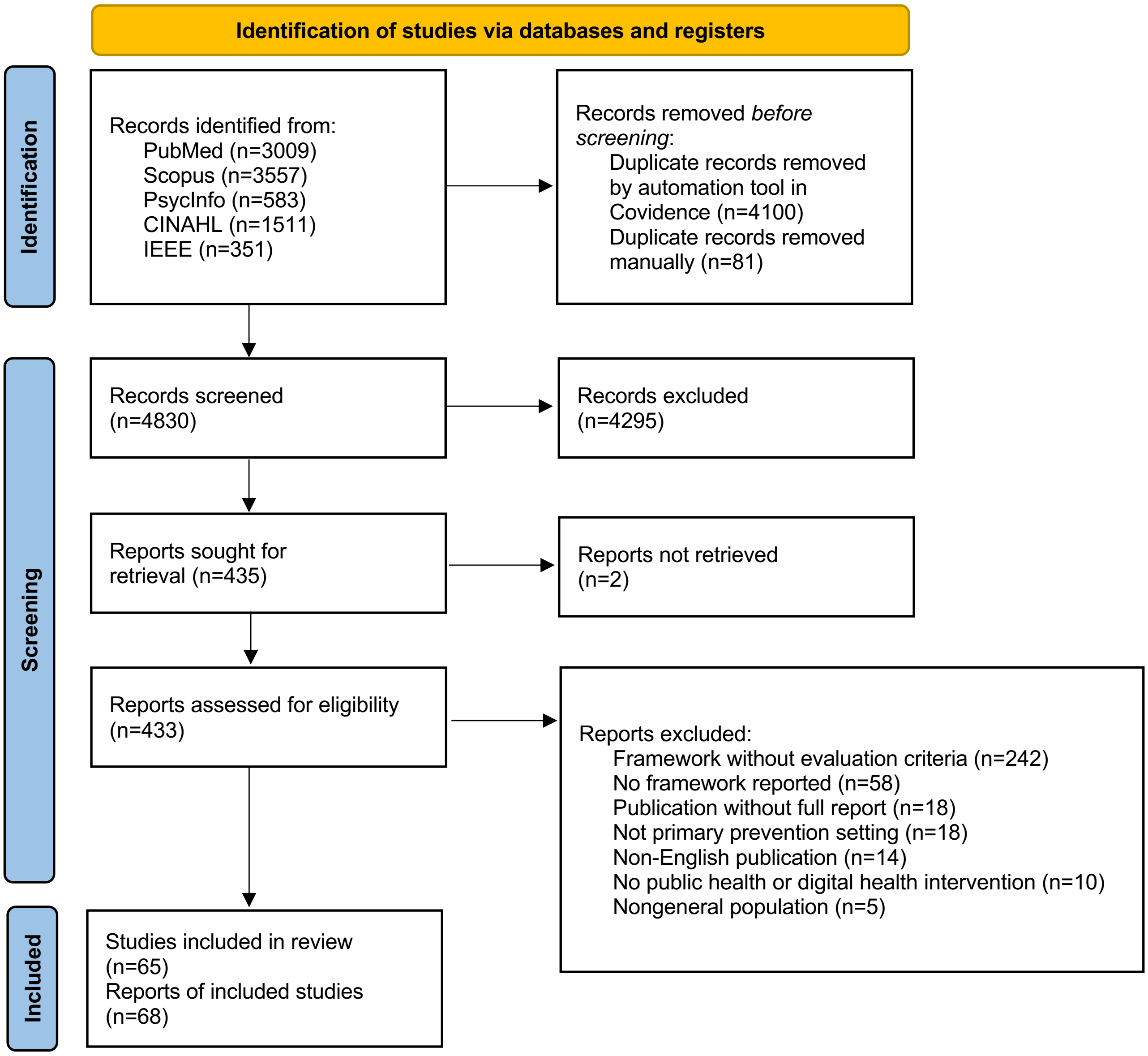


*From:* Page MJ, McKenzie JE, Bossuyt PM, Boutron I, Hoffmann TC, Mulrow CD, et al. The PRISMA 2020 statement: an updated guideline for reporting systematic reviews. BMJ 2021;372:n71. doi: 10.1136/bmj.n71

For more information, visit: http://www.prisma-statement.org/
