# Supplementary material for: Developing and Evaluating Digital Public Health Interventions Using the Digital Public Health Framework DigiPHrame: A Framework Development Study
Source: J Med Internet Res. 2024 Sep 12;26:e54269. doi: 10.2196/54269 (PMC11427851; doi:10.2196/54269)
Supplement: Multimedia Appendix 3 [file jmir_v26i1e54269_app3.docx]

| **HTA** | **Framework before consensus meeting** | **Framework**  **Version 1** | **Framework**  **Version 2 (current)** |
| --- | --- | --- | --- |
| Health Problem   - Target population - Target condition - Current Management of the Condition - Utilisation - Regulatory Status - None of the above | - Health Condition and current Public Health interventions - Population - Conditions - Health Inequities - Current Public Health Interventions | - Health Conditions & Current Public Health Interventions (14) - Population (5) - Conditions (4) - Health Inequities (2) - Current Public Health Interventions (3) | - Health Conditions & Current Public Health Interventions (16)   - Population (5)  - Conditions (4)  - Health Inequities (4)  - Current Public Health Interventions (3) |
| Description and technical   - Features of the technology - Regulatory Status - Investments and tools required to use the technology - Training and information needed to use the technology - None of the above | - Functionality of Technology - Features - Intervention Design - Evidence-Based Content - Technical Properties - Launch - Update - Provider - Interoperability - Data integration - Error rates - Accuracy - Scalability - Internet Connectivity - Data usage - Design quality - Human-computer Interaction - User-friendliness/Usability - User satisfaction - Rating - Co-creation - Language - Feasibility - Capacity Building - Training - Users’ Perspective - Accessibility - Usability - User’s needs - Credibility/trustfulness - Adaptability - Empowerment/self-efficacy - Participation | - Functionality of the Health Technologies (9) - Features (3) - Intervention Design (6) - Software properties (18) - Launch (2) - Update (2) - Rating (1) - Provider (1) - Interoperability (3) - Data integration (2) - Open Source (2) - Stability (1) - Internet Connectivity (4) - Human-Computer Interaction (20) - Accessibility (2) - Languages (2) - User-friendliness (4) - Usability (6) - Co-creation and Empowerment (3) - Credibility & Trustfulness (1) - Feasibility (1) - Design Quality (1) | - Technical Aspects (21) - Functionality (5) - Interoperability (4) - Data integration (2) - Open-Source (4) - Stability (1) - Internet Connectivity (4) - Feasibility (1) - Usability (19) - Accessibility (4) - (& Languages) - Usability (10) - Co-creation and   Empowerment (3)   - Credibility &Trustfulness (2) |
| Safety   - Patient safety - Occupational safety - Environmental safety - Safety risk management - None of the above | - Data Security and Data Autonomy - Data Confidentiality - Data Integrity - Data Authenticity - Data Availability - Data Controllability - Data Autonomy | - Data Security and Data Protection (28) - Data Confidentiality (4) - Data Integrity (3) - Data Authenticity (2) - Data Availability (5) - Data Controllability (3) - Data Protection (11) | - Data Security and Data Protection (30) - Data Confidentiality (4) - Enforcement of Data Integrity (3) - Data Authenticity (2) - Data Availability (5) - Data Controllability (2) - Data Protection (14) |
| Clinical Effectiveness   - Mortality - Morbidity - Function - Health- related quality of life - Quality of life - Patient satisfaction - Test- treatment chain - Test accuracy - Patient safety - Change-in management - Benefit- harm balance - None of the above | - Health - Mortality - Effects on Health - Function - Quality of Life and Well-being - Behaviour Change - User satisfaction - Negative Effects - Individuals and the third parties - Environmental - Equity - Data Security - Social and Political | - Health-related Effects (15) - Mortality (1) - Effects on Health (1) - Function (1) - Quality of Life and Well-being (3) - Knowledge and Behaviour Change (9) | - Intended and Unintended Health-Related Effects (15) - Mortality (1) - Health Outcome (1) - Function (1) - Quality of Life and Well-being (3) - Knowledge and Behaviour Change (9) |
| Costs and economic   - Resource utilisation - Measurement and estimation of outcomes - Examination of costs and outcomes - Characterising uncertainty - Characterising heterogeneity - Validity of the model(s) - None of the above | - Cost and Economics - Measurement of outcomes - Estimation of resources and costs - Relating costs and outcomes - Financing | - Cost and Economics (6) - Prior to the Economic Assessment (3) - Economic Evaluation Methods Potentially Relevant (3) | - Cost and Economics (7) - Prior to the Economic   Assessment (3)   - Economic Evaluation Methods Potentially Relevant (4) |
| Ethics   - Benefit- harm balance - Autonomy - Respect for persons - Justice and Equity - Legislation - Ethical consequences of the HTA - None of the above | - Ethics - Autonomy - Harm/non-maleficience - Beneficence - Justice and equity - Balance | - Ethics (26) - Autonomy (9) - Harm/non-maleficience (7) - Beneficience (3) - Justice (7) | - Ethics (24) - Autonomy (7) - Harm/non-maleficience (7) - Beneficience (3) - Justice (7) |
| Organizational aspects   - Health delivery process - Structure of health care system - Process- related costs - Management - Culture - None of the above | - Infrastructure and Organization - Structure of the setting - Infraestructure - Inter-organizational relationship - Intervention consequences on the health system | - Infrastructure and Organization (17) - Structure of the setting (3) - Infraestructure (3) - Inter-organizational relationship (7) - Health System Interaction (4) | - Infrastructure and Organization (11) - Infraestructure of the context (4) - Intra-organizational relationship (5) - Health System Interaction (2) |
| Patients and social aspects   - Patients' perspectives - Social group aspects - Communication aspects - None of the above | - Social and Cultural Aspects - Social and societal impact - Socio-cultural Acceptability - Social sustainability - Community Capacity - Community Participation | - Social, Cultural & Gender Aspects (12) - Context/Setting (1) - Social and societal impact (1) - Impact on societal groups (1) - Impact related to gender (1) - Socio-cultural Acceptability (1) - Social sustainability (3) - Community Capacity (3) - Community Participation (1) | - Social, Cultural & Intersectional Aspects (6) - Context (1) - Social and societal impact (1) - Impact on Intersectional Social Positions (1) - Socio-cultural Acceptability (1) - Community Capacity (1) - Community Participation (1) |
| Legal aspects   - Autonomy of the patient - Privacy of the patient - Equality in health care - Ethical aspects - Authorisation and safety - Ownership and liability - Regulation of the market - None of the above | - Legal and Regulatory - Data protection - Data security - Consumer Protection - Medical (Device) - Health Insurances | - Legal and Regulatory (13) - Data protection (7) - Data security (1) - Consumer Protection (1) - Medical (Device) (3) - Health System Financing (1) | - Legal and Regulatory (6) - Data protection (1) - Data security (1) - Consumer Protection (1) - Medical (Device) (2) - Health System Financing (1) |
|  | - Sustainability - Environmental Sustainability - Social Sustainability - Economic Sustainability | - Sustainability (9) - Environmental Sustainability (3) - Social Sustainability (4) - Economic Sustainability (2) | - Sustainability (8) - Environmental Sustainability (4) - Social Sustainability (2) - Economic Sustainability (2) |
|  | - Implementation - Implementation theory - Implementation structure - Implementation process - Implementation Strategy - Implementation Agent - Implementation Outcome   Complexity (Practical Implementation Difficulties | - Implementation (23) - Implementation theory (3) - Implementation structure (8) - Implementation process (3) - Implementation Strategy (3) - Implementation Agent (3) - Implementation Outcome (2) - Complexity (Practical Implementation Difficulties) (1) | - Implementation (18) - Implementation theory (2) - Implementation Infrastructure (6) - Implementation Process (4) - Implementation Agent (1) - Implementation Outcome (2) - Dissemination (3) |
