# Supplementary material for: Developing and Evaluating Digital Public Health Interventions Using the Digital Public Health Framework DigiPHrame: A Framework Development Study
Source: J Med Internet Res. 2024 Sep 12;26:e54269. doi: 10.2196/54269 (PMC11427851; doi:10.2196/54269)
Supplement: Multimedia Appendix 4 [file jmir_v26i1e54269_app4.docx]

# Articles included for data extraction

Framework for program evaluation in public health. (1999). MMWR Recomm Rep, 48(Rr-11), 1-40.

Aguirre, R. T. P., McCoy, M. K., & Roan, M. (2013). Development Guidelines from a Study of Suicide Prevention Mobile Applications (Apps). Journal of Technology in Human Services, 31(3), 269-293. https://doi.org/10.1080/15228835.2013.814750

Akrami, F., Zali, A., Abbasi, M., Majdzadeh, R., Karimi, A., Fadavi, M., & Mehrabi Bahar, A. (2018). An ethical framework for evaluation of public health plans: a systematic process for legitimate and fair decision-making. Public Health, 164, 30-38. https://doi.org/10.1016/j.puhe.2018.07.018

Bryant, C. A., Courtney, A. H., McDermott, R. J., Lindenberger, J. H., Swanson, M. A., Mayer, A. B., Panzera, A. D., Khaliq, M., Schneider, T., Wright, A. P., Craig Lefebvre, R., & Biroscak, B. J. (2014). Community - Based prevention marketing for policy development: A new planning framework for coalitions. Social Marketing Quarterly, 20(4), 219-246. https://doi.org/10.1177/1524500414555948

Bunde-Birouste, A. W., & Ritchie, J. E. (2007). Strengthening peace-building through health promotion development of a framework. Global Perspectives on Health Promotion Effectiveness, 247-258. https://doi.org/10.1007/978-0-387-70974-1_15

Chan, S., Torous, J., Hinton, L., & Yellowlees, P. (2015). Towards a Framework for Evaluating Mobile Mental Health Apps. Telemed J E Health, 21(12), 1038-1041. https://doi.org/10.1089/tmj.2015.0002

Cunningham, F. C., Ranmuthugala, G., Westbrook, J. I., & Braithwaite, J. (2019). Tackling the wicked problem of health networks: the design of an evaluation framework. BMJ Open, 9(5), e024231. https://doi.org/10.1136/bmjopen-2018-024231

Eslava-Schmalbach, J., Garzón-Orjuela, N., Elias, V., Reveiz, L., Tran, N., & Langlois, E. V. (2019). Conceptual framework of equity-focused implementation research for health programs (EquIR). Int J Equity Health, 18(1), 80. https://doi.org/10.1186/s12939-019-0984-4

EUnetHTA Joint Action 2, Work Package 8. HTA Core Model ® version 3.0 (Pdf); 2016. Available from www.htacoremodel.info/BrowseModel.aspx.

Fadzillah, F. M., & Arshad, N. I. (2016). Evaluating the impact of non-medical m-health application: Towards development of a framework. 137-142. https://doi.org/10.1109/ICCOINS.2016.7783203

Fanta, G. B., & Pretorius, L. (2018). A conceptual framework for sustainable ehealth implementation in resource-constrained settings. South African Journal of Industrial Engineering, 29(3 Special Edition), 132-147. https://doi.org/10.7166/29-3-2055

Fanta, G. B., Pretorius, L., & Erasmus, L. (2015). An evaluation of ehealth systems implementation frameworks for sustainability in resource constrained environments: A literature review. 1046-1063. https://www.scopus.com/inward/record.uri?eid=2-s2.0-84975747544&partnerID=40&md5=a35f261ab9c9f687fc9c353990aa4254

Glasgow, R. E., Harden, S. M., Gaglio, B., Rabin, B., Smith, M. L., Porter, G. C., Ory, M. G., & Estabrooks, P. A. (2019). RE-AIM Planning and Evaluation Framework: Adapting to New Science and Practice With a 20-Year Review. Front Public Health, 7, 64. https://doi.org/10.3389/fpubh.2019.00064

Glasgow, R. E., Vogt, T. M., & Boles, S. M. (1999). Evaluating the public health impact of health promotion interventions: the RE-AIM framework. Am J Public Health, 89(9), 1322-1327. https://doi.org/10.2105/ajph.89.9.1322

Gopichandran, V., & Indira Krishna, A. K. (2013). Monitoring 'monitoring' and evaluating 'evaluation': an ethical framework for monitoring and evaluation in public health. J Med Ethics, 39(1), 31-35. https://doi.org/10.1136/medethics-2012-100680

Green, L. W. (1986). Evaluation model: a framework for the design of rigorous evaluation of efforts in health promotion. Am J Health Promot, 1(1), 77-79. https://doi.org/10.4278/0890-1171-1.1.77

Hamid, A., & Sarmad, A. (2009). Towards an evaluation framework for e-health services: Evaluating criteria from users perspective. Handbook of Research on Advances in Health Informatics and Electronic Healthcare Applications: Global Adoption and Impact of Information Communication Technologies, 1-16. https://doi.org/10.4018/978-1-60566-030-1.ch001

Henson, P., David, G., Albright, K., & Torous, J. (2019). Deriving a practical framework for the evaluation of health apps. Lancet Digit Health, 1(2), e52-e54. https://doi.org/10.1016/s2589-7500(19)30013-5

Huff, R. M., Kline, M. V., & Visencio Dobbins, D. (2015). The Cultural Assessment Framework. Huff, Robert M [Ed]; Kline, Michael V [Ed]; Peterson, Darleen V [Ed] (2015) Health promotion in multicultural populations: A handbook for practitioners and students , 3rd ed (pp 127-149) xxi, 534 pp Thousand Oaks, CA, US: Sage Publications, Inc; US, 127-149. https://ovidsp.ovid.com/ovidweb.cgi?T=JS&CSC=Y&NEWS=N&PAGE=fulltext&D=psyc12&AN=2014-20479-006

Jolley, G., Lawless, A., & Hurley, C. (2008). Framework and tools for planning and evaluating community participation, collaborative partnerships and equity in health promotion. Health Promot J Austr, 19(2), 152-157. https://doi.org/10.1071/he08152

Karni, L., Dalal, K., Memedi, M., Kalra, D., & Klein, G. O. (2020). Information and Communications Technology-Based Interventions Targeting Patient Empowerment: Framework Development. J Med Internet Res, 22(8), e17459. https://doi.org/10.2196/17459

Khoja, S., Durrani, H., Scott, R. E., Sajwani, A., & Piryani, U. (2013). Conceptual framework for development of comprehensive e-health evaluation tool. Telemed J E Health, 19(1), 48-53. https://doi.org/10.1089/tmj.2012.0073

Kiberu, V. M., Mars, M., & Scott, R. E. (2021). Development of an evidence-based e-health readiness assessment framework for Uganda. Health Inf Manag, 50(3), 140-148. https://doi.org/10.1177/1833358319839253

Klepac Pogrmilovic, B., O'Sullivan, G., Milton, K., Biddle, S. J. H., Bauman, A., Bellew, W., Cavill, N., Kahlmeier, S., Kelly, M. P., Mutrie, N., Pratt, M., Rutter, H., Ramirez Varela, A., Woods, C., & Pedisic, Z. (2019). The development of the Comprehensive Analysis of Policy on Physical Activity (CAPPA) framework. Int J Behav Nutr Phys Act, 16(1), 60. https://doi.org/10.1186/s12966-019-0822-5

Lafferty, C. K., & Mahoney, C. A. (2003). A framework for evaluating comprehensive community initiatives. Health Promot Pract, 4(1), 31-44. https://doi.org/10.1177/1524839902238289

Lagan, S., Emerson, M. R., King, D., Matwin, S., Chan, S. R., Proctor, S., Tartaglia, J., Fortuna, K. L., Aquino, P., Walker, R., Dirst, M., Benson, N., Myrick, K. J., Tatro, N., Gratzer, D., & Torous, J. (2021). Mental Health App Evaluation: Updating the American Psychiatric Association's Framework Through a Stakeholder-Engaged Workshop. Psychiatr Serv, 72(9), 1095-1098. https://doi.org/10.1176/appi.ps.202000663

Lagan, S., Sandler, L., & Torous, J. (2021). Evaluating evaluation frameworks: a scoping review of frameworks for assessing health apps. BMJ Open, 11(3), e047001. https://doi.org/10.1136/bmjopen-2020-047001

Leeman, J., Sommers, J., Vu, M., Jernigan, J., Payne, G., Thompson, D., Heiser, C., Farris, R., & Ammerman, A. (2012). An evaluation framework for obesity prevention policy interventions. Prev Chronic Dis, 9, E120. https://doi.org/10.5888/pcd9.110322

León-Castañeda, C. D. (2019). Electronic health (e-Health): A conceptual framework for implementation in health services. Gaceta Medica de Mexico, 155(2), 176-183. https://www.scopus.com/inward/record.uri?eid=2-s2.0-85065654571&partnerID=40&md5=5c2a2fa1718a09d63190857bfbd0dd47

Mayoka, K. G., Rwashana, A. S., Mbarika, V. W., & Isabalija, S. (2012). A framework for designing sustainable telemedicine information systems in developing countries. Journal of Systems and Information Technology, 14(3), 200-219. https://doi.org/10.1108/13287261211255329

Mburu, S., Franz, E., & Springer, T. (2013). A conceptual framework for designing mHealth solutions for developing countries. 31-36. https://doi.org/10.1145/2491148.2491154

McIntosh, E., & Cairns, J. (1997). A framework for the economic evaluation of telemedicine. J Telemed Telecare, 3(3), 132-139. https://doi.org/10.1258/1357633971931039

McLees, A. W., Nawaz, S., Thomas, C., & Young, A. (2015). Defining and assessing quality improvement outcomes: A framework for public health. American Journal of Public Health, 105, S167-S173. https://doi.org/10.2105/AJPH.2014.302533

Noar, S. M. (2012). An Audience-Channel-Message-Evaluation (ACME) Framework for Health Communication Campaigns. Health Promotion Practice, 13(4), 481-488. https://doi.org/10.1177/1524839910386901

O'Connor-Fleming, M. L., Parker, E., Higgins, H., & Gould, T. (2006). A framework for evaluating health promotion programs. Health Promot J Austr, 17(1), 61-66. https://doi.org/10.1071/he06061

Paryani, S. (2006). A framework for evaluation of telemedicine. 202-205. https://doi.org/10.1109/health.2006.246450

Pfadenhauer, L. M., Gerhardus, A., Mozygemba, K., Lysdahl, K. B., Booth, A., Hofmann, B., Wahlster, P., Polus, S., Burns, J., Brereton, L., & Rehfuess, E. (2017). Making sense of complexity in context and implementation: the Context and Implementation of Complex Interventions (CICI) framework. Implement Sci, 12(1), 21. https://doi.org/10.1186/s13012-017-0552-5

Poland, B., Krupa, G., & McCall, D. (2009). Settings for health promotion: an analytic framework to guide intervention design and implementation. Health Promot Pract, 10(4), 505-516. https://doi.org/10.1177/1524839909341025

Ramos, G., Ponting, C., Labao, J. P., & Sobowale, K. (2021). Considerations of diversity, equity, and inclusion in mental health apps: A scoping review of evaluation frameworks. Behav Res Ther, 147, 103990. https://doi.org/10.1016/j.brat.2021.103990

Sadegh, S. S., Khakshour Saadat, P., Sepehri, M. M., & Assadi, V. (2018). A framework for m-health service development and success evaluation. Int J Med Inform, 112, 123-130. https://doi.org/10.1016/j.ijmedinf.2018.01.003

Sharma, S., Gergen Barnett, K., Maypole, J. J., & Grochow Mishuris, R. (2022). Evaluation of mHealth Apps for Diverse, Low-Income Patient Populations: Framework Development and Application Study. JMIR Form Res, 6(2), e29922. https://doi.org/10.2196/29922

Stead, M., Hastings, G., & Eadie, D. (2002). The challenge of evaluating complex interventions: a framework for evaluating media advocacy. Health Educ Res, 17(3), 351-364. https://doi.org/10.1093/her/17.3.351

Stratil, J. M., Baltussen, R., Scheel, I., Nacken, A., & Rehfuess, E. A. (2020). Development of the WHO-INTEGRATE evidence-to-decision framework: an overview of systematic reviews of decision criteria for health decision-making. Cost Eff Resour Alloc, 18, 8. https://doi.org/10.1186/s12962-020-0203-6

Tarricone, R., Petracca, F., Cucciniello, M., & Ciani, O. (2022). Recommendations for developing a lifecycle, multidimensional assessment framework for mobile medical apps. Health Econ. https://doi.org/10.1002/hec.4505

Thurston, W. E., Vollman, A. R., Wilson, D. R., MacKean, G., Felix, R., & Wright, M. F. (2003). Development and testing of a framework for assessing the effectiveness of health promotion. Soz Praventivmed, 48(5), 301-316. https://doi.org/10.1007/s00038-003-2057-z

van Weert, J. C. M., Hermanns, S. S. T., Linn, A. J., & Schouten, B. C. (2011). Dance4life: evaluating a global HIV and AIDS prevention program for young people using the Pre-Im framework for process evaluation. International Public Health Journal, 3(1), 99-110. https://search.ebscohost.com/login.aspx?direct=true&db=cin20&AN=104849313&site=ehost-live

Vanderkruik, R., & McPherson, M. E. (2017). A Contextual Factors Framework to Inform Implementation and Evaluation of Public Health Initiatives. American Journal of Evaluation, 38(3), 348-359. https://doi.org/10.1177/1098214016670029

Wienert, J., & Zeeb, H. (2021). Implementing Health Apps for Digital Public Health - An Implementation Science Approach Adopting the Consolidated Framework for Implementation Research. Front Public Health, 9, 610237. https://doi.org/10.3389/fpubh.2021.610237

Wolfenstetter, S. B. (2011). Conceptual framework for standard economic evaluation of physical activity programs in primary prevention. Prev Sci, 12(4), 435-451. https://doi.org/10.1007/s11121-011-0235-4

Zadey, S., Dharmadhikari, S., & Mukuntharaj, P. (2021). Ethics-driven policy framework for implementation of movement restrictions in pandemics. BMJ Glob Health, 6(6). https://doi.org/10.1136/bmjgh-2021-005202

Zelmer, J., van Hoof, K., Notarianni, M., van Mierlo, T., Schellenberg, M., & Tannenbaum, C. (2018). An Assessment Framework for e-Mental Health Apps in Canada: Results of a Modified Delphi Process. JMIR Mhealth Uhealth, 6(7), e10016. https://doi.org/10.2196/10016
